# Long-term outcome of postpartum psychosis: a prospective clinical cohort study in 106 women

**DOI:** 10.1186/s40345-021-00236-2

**Published:** 2021-10-28

**Authors:** Anna-Sophie Rommel, Nina Maren Molenaar, Janneke Gilden, Steven A. Kushner, Nicola J. Westerbeek, Astrid M. Kamperman, Veerle Bergink

**Affiliations:** 1 Department of Psychiatry, Icahn School of Medicine at Mount Sinai, Icahn (East) Building Floor 4 Room 34, 1425 Madison Ave, New York, NY 10029 USA; 2 Department of Obstetrics, Gynecology and Reproductive Science, Icahn School of Medicine at Mount Sinai, New York, NY USA; 3 Department of Psychiatry, Erasmus Medical Center Rotterdam, Rotterdam, The Netherlands

**Keywords:** Postpartum psychosis, Bipolar disorder, Longitudinal follow-up, Course of illness, Clinical predictors

## Abstract

**Objective:**

We aimed to investigate the outcome of postpartum psychosis over a four-year follow-up, and to identify potential clinical markers of mood/psychotic episodes outside of the postpartum period.

**Methods:**

One hundred and six women with a diagnosis of first-onset mania or psychosis during the postpartum period were included in this prospective longitudinal study. Women were categorized into either (1) recurrence of non-postpartum mood/psychotic episodes or (2) mania/psychosis limited to the postpartum period. We summarize the longitudinal course of the illness per group. We used a logistic regression model to identify clinical predictors of recurrence of mood/psychotic episodes outside of the postpartum period.

**Results:**

Over two thirds of the women included in this study did not have major psychiatric episodes outside of the postpartum period during follow-up. The overall recurrence rate of mood/psychotic episodes outside the postpartum period was ~ 32%. Of these women, most transitioned to a bipolar disorder diagnosis. None of the women fulfilled diagnostic criteria for schizophrenia or schizophreniform disorder. No clinical markers significantly predicted recurrence outside of the postpartum period.

**Conclusions:**

For the majority of women with first-onset postpartum psychosis, the risk of illness was limited to the period after childbirth. For the remaining women, postpartum psychosis was part of a mood/psychotic disorder with severe non-postpartum recurrence, mainly in the bipolar spectrum. No clinical predictors for risk of severe episodes outside the postpartum period emerged. Our findings add to previous evidence suggesting a fundamental link between postpartum psychosis and bipolar disorder, which may represent two distinct diagnoses within the same spectrum.

**Supplementary Information:**

The online version contains supplementary material available at 10.1186/s40345-021-00236-2.

## Background

Postpartum psychosis is an umbrella term for postpartum mania, psychosis, psychotic depression and a mixed affective state, occurring shortly after childbirth (Bergink et al. 2011; Kapfhammer et al. 2014; Osborne 2018). Postpartum psychosis is the most severe form of childbirth-related psychiatric disorders and has an incidence of ~ 0.3 to 0.6 per 1000 births (Bergink et al. 2011,2015a; Kapfhammer et al. 2014; Meltzer-Brody et al. 2018). Women with postpartum psychosis may initially present with mood fluctuations, insomnia and obsessive concerns about the baby, followed by severe mood symptoms, and sometimes disorganized behavior, delusions and hallucinations (Bergink et al. 2011; Kamperman et al. 2017; Brockington et al. 1981; Boyce and Barriball 2010; Sit et al. 2006). The presence of severe mood symptoms differentiates postpartum psychosis from psychosis outside of the postpartum period (Bergink et al. 2016). Postpartum psychosis is, therefore, a misnomer. Since psychotic symptoms in the postpartum period occur mostly within the setting of affective lability, the disorder is a bipolar-related mood disorder rather than a primary psychotic disorder (Meltzer-Brody et al. 2018; Bergink et al. 2015a).

Due to the high relative risk for suicide and infanticide, early recognition and adequate treatment of postpartum psychosis is crucial (Bergink et al. 2011; Wisner et al. 1994). With adequate treatment, nearly all women with postpartum psychosis achieve full remission (Bergink et al. 2015b), and a large proportion of patients achieve good functional recovery (Burgerhout et al. 2017). For some women, postpartum psychosis is part of a severe, often life-long, psychiatric disorder (Bergink et al. 2016; Munk-Olsen et al. 2012; Chaudron and Pies 2003; Nager et al. 2013). For other women, the vulnerability is limited to the postpartum period (Bergink et al. 2016; Wesseloo et al. 2016).

Despite the widespread use of the term ‘postpartum psychosis’, this diagnosis is not recognized in current classification systems, including the International Classification of Diseases, Tenth Revision (ICD-10) and the Diagnostic and Statistical Manual of Mental Disorders, Fifth Edition (DSM-5) (Florio et al. 2016). Instead, the majority of women with postpartum psychosis receive a DSM-5 diagnosis of bipolar disorder, because they present with prominent manic or mixed affective episodes. Yet, according to our recent meta-analysis, 43.5% of women with postpartum psychosis have no manic or psychotic recurrence outside the postpartum period over a mean follow-up of 16 years (Gilden et al. 2020), suggesting that a diagnosis of bipolar disorder might not always be warranted. It is important to note that most studies included in the meta-analysis were performed in the 1970s and 1980s, limiting the generalizability of the results. Further research is needed to reproduce these numbers in the current treatment setting. In addition, to improve long-term prognosis, it is pertinent to identify those women who may develop severe mood episodes outside the postpartum period.

Currently, little is known about which women are specifically at risk for recurrence outside the postpartum period. Previous studies identified being single/unmarried (Terp et al. 1999), a personal or family history of psychiatric disorders and older age (Blackmore et al. 2013) as potential risk factors for future recurrence after first onset postpartum psychosis (Kapfhammer et al. 2014; Benvenuti et al. 1992). However, these studies were small and conducted retrospectively. Consequently, this prospective longitudinal study was designed to investigate recurrence in 106 women with postpartum psychosis over a four-year period. We further aimed to identify potential clinical markers of a psychiatric disorder with mood or psychotic episodes outside of the postpartum period.

## Methods

### Study setting and procedure

The study was approved by the International Review Board of the Erasmus Medical Centre (Rotterdam, The Netherlands). All patients provided written informed consent. The study was performed on the Mother-Baby Unit (MBU), a five-bed inpatient unit that specializes in the care of patients with severe psychopathology in the postpartum period, located in the Department of Psychiatry in the Erasmus Medical Centre (Erasmus MC) in Rotterdam, The Netherlands. On the MBU, women are admitted with their babies, who stay in a fully staffed nursery adjoining the unit (Bergink et al. 2011). Every patient admitted to the MBU between May 2005 and December 2016 was screened for study inclusion (N = 315).

### Participants

We included patients with a diagnosis of first-onset mania or psychosis during the postpartum period, who were aged between 18 and 45 years. ‘Postpartum psychosis’ was operationalized as any of the following DSM-IV diagnoses and requiring the specifier ‘onset postpartum’: manic episode, mixed episode, depressive disorder with psychotic features, psychotic disorder not otherwise specified (NOS) or brief psychotic disorder, as assessed with the Structured Clinical Interview for DSM (SCID). Patients were excluded if they had a chronic psychotic disorder, mania, or psychosis with onset during pregnancy or > 12 weeks postpartum, a history of psychosis or mania outside the postpartum period, or drug abuse.

A total of 315 women were admitted to the MBU between May 2005 and December 2016. One hundred thirty-seven of these patients received a diagnosis of postpartum psychosis. Of these, 14 women had a prior postpartum psychiatric episode but no episodes of mania or psychosis at other times. Of the 137 women, four patients declined participation. In addition, 21 women were excluded: 18 women were excluded because they had a history of mania or psychosis outside the postpartum period, one woman was excluded because of postpartum drug abuse, one woman was excluded because her symptom onset was > 12 weeks postpartum, one woman was excluded because her symptoms started during pregnancy. Accordingly, 112 patients fulfilled the criteria for first-onset postpartum psychosis. Five patients were lost to follow-up (4.5%) and one patient (0.9%) was lost to suicide (baseline and clinical characteristics of these women can be found in Additional file 1: Table A1). In this study, we therefore included 106 women admitted to the MBU between 2005 and 2016. None of the women were breastfeeding during their MBU admission.

### Symptomatology and clinical course of the initial episode

Patients were diagnosed by a clinician using the Structured Clinical Interview for DSM (SCID-1/P research version) (First et al. 1999). The SCID is a semi-structured interview guide for making diagnoses according to the diagnostic criteria published in the American Psychiatric Association’s Diagnostic and Statistical Manual for Mental Disorders (DSM). Previous hypomanic and manic episodes were also registered using the SCID. We further assessed demographics, psychiatric history, and family history of psychiatric illness (Table 1) (for more detail, see 12).

**Table 1:** Demographics and clinical characteristics of women with non-postpartum recurrence during follow-up and of women with no recurrence outside the postpartum period during follow-up

	Non-postpartum recurrence n = 34		No recurrence outside the postpartum period n = 72		p-value
Baseline characteristics at time of initial episode					
Age in years	Mean = 31.0	SD = 4.8	Mean = 31.9	SD = 4.9	0.351
Country of origin (n)					
Netherlands	79.4%	27	94.4%	68	0.018
Other	20.6%	7	5.6%	4	
Marital status (n)					
Married or in relationship	100.0%	34	91.6%	66	0.217
Not in relationship	-	-	4.2%	3	
Missing	-	-	4.2%	3	
Education (n)					
No education	3.0%	1	–	–	0.521
Primary school	–	–	1.4%	1	
Secondary school	14.7%	5	9.7%	7	
Vocational training	29.4%	10	30.6%	22	
Higher education	52.9%	18	58.3%	42	
Parity (n)					
1	79.4%	27	79.2%	57	0.343
2	11.8%	4	16.7%	12	
≥ 3	8.8%	3	2.8%	2	
Missing	–	–	1.4%	1	
Psychiatric history before postpartum episode (n)					
None	55.9%	19	76.4%	55	0.210
Postpartum depression	8.8%	3	2.8%	2	
Postpartum psychosis/mania (not at other times)	8.8%	3	5.6%	4	
Depression	20.6%	7	9.7%	7	
Anxiety	5.9%	2	2.8%	2	
Hypomania	–	–	2.8%	2	
Family history of psychiatric disorders^†^ (n)					
None	47.1%	16	34.7%	25	0.238
1^st^ degree relative with depression or anxiety	41.2%	14	36.1%	26	0.616
1^st^ degree relative with postpartum psychiatric episode	2.9%	1	13.9%	10	0.085
1^st^ degree relative with bipolar disorder	8.8%	3	13.9%	10	0.444
Missing	2.9%	1	4.2%	3	0.745
Length of initial hospital admission in days	Mean = 59.6	SD = 25.6	Mean = 57.3	SD = 31.2	0.700
Phenomenology initial episode (n)					
Manic with and without psychotic features	58.8%	20	61.1%	44	0.533
Psychotic only	17.7%	6	12.5%	9	
Depressed-psychotic	5.8%	2	13.9%	10	
Manic-depressed (mixed)	17.7%	6	12.5%	9	
Relation between mood and psychotic symptoms (n)					
Presence of mood-incongruent psychotic symptoms	67.6%	23	62.5%	45	0.611
> 50% of time psychotic during initial episodes	52.9%	18	47.2%	34	0.596
First rank psychotic symptoms^‡^	8.8%	3	6.9%	5	0.730
DSM-IV diagnosis at baseline					
Bipolar I disorder	82.3%	28	77.8%	56	0.600
Bipolar II disorder	2.9%	1	1.4%	1	0.597
Major Depressive Disorder with Psychotic Features	2.9%	1	11.1%	8	0.159
Mood disorders NOS	8.8%	3	9.7%	7	0.883
Lithium treatment during admission (n)					
No	20.6%	7	25.0%	18	0.617
Yes	79.4%	27	75.0%	54	
Antipsychotics treatment during admission (n)					
No	14.7%	5	18.1%	13	0.668
Yes	85.3%	29	81.1%	59	
Follow-up					
Length of follow-up period in months	Mean = 46.3	SD = 20.3	Mean = 44.5	SD = 8.8	0.543
Subsequent pregnancies (n)					
No	64.7%	22	58.3%	42	0.464
Yes	32.3%	11	40.3%	29	
Missing	3.0%	1	1.4%	1	
Recurrence period (n)					
Postpartum only	–	–	3.0	2	< 0.001
Non-postpartum only	90.9%	30	-	-	
Postpartum and non-postpartum	9.1%	3	–	–	
Recurrence phenomenology (n)					
No recurrence	–	–	97.2%	70	< 0.001
Hypo(mania)	42.4%	13	–		
Depression/Anxiety	33.3%	11	2.8*%	2*	
Psychotic episode without affective components	15.2%	5	–	–	
Schizoaffective disorder	9.1%	4	–	–	
DSM-IV diagnosis at follow-up					
Bipolar I Disorder	38.2%	13	4.2%	3	< 0.001
Major Depressive Disorder	14.7%	5	16.7%	12	0.794
Anxiety/Panic Disorder	11.8%	4	2.8%	2	0.063
Brief Psychotic Disorder	11.8%	4	-	-	< 0.001
Psychotic Disorder NOS	2.9%	1	-	-	< 0.001
Schizoaffective Disorder	11.8%	4	–	–	< 0.001
Mood Disorder NOS	2.9%	1	–	–	< 0.001
Cyclothymic Disorder	2.9%	1	–	–	< 0.001
Observation of other suspected mental condition (V71.09)	2.9%	1	75%	54	< 0.001
Lithium stop in follow-up period (n)					
No	61.8%	21	45.8%	33	0.126
Yes	38.2%	13	54.2%	39	
Recurrence *within 6 months after* lithium stop (n)					
No	23.5%	8	–	–	< 0.001
Yes	14.7%	5	–	–	
Lithium treatment at follow-up (n)					
No	38.2%	13	61.1%	44	0.027
Yes	61.8%	21	38.9%	28	
Antipsychotics treatment at follow-up (n)					
No	76.5%	26	94.4%	68	0.006
Yes	23.5%	8	5.6%	4	
Still in treatment at follow-up (n)					
No	20.6%	7	68.0%	49	< 0.001
Yes	76.5%	26	30.6%	22	
Missing	2.9%	1	1.4%	1	

The table presents percentages and numbers of participants, unless stated

^†^Percentages may exceed 100% because these categories are not mutually exclusive

^‡^The set of psychotic symptoms recognized as having special weight in the diagnosis of schizophrenia and schizoaffective disorder

*Recurrence in the postpartum period

Phenomenology of the initial episode was assessed using the Bipolar Affective Disorder Dimension Scale (BADDS) (Craddock et al. 2004). The BADDS comprises four dimensions which provide a quantitative measure of psychopathology in each of four domains: (1) Manic-like episodes (the Mania dimension, M), (2) Depression-like episodes (the Depression dimension, D), (3) Psychotic symptomatology (the Psychosis dimension, P) and (4) the relationship (congruence of content and timing) between psychotic features (if present) and mood episodes (the Incongruence dimension, I). Each dimension provides a composite measure that takes both severity and frequency of relevant psychopathology into account. The dimensions are rated using integers in the range 0–100, with higher scores indicating more clinically important psychopathology—typically a mix of severity and frequency/duration.

### Treatment regimen

Because clinical presentation, family history, and the longitudinal illness course overlap markedly with those of bipolar disorder, postpartum psychosis is generally considered a bipolar spectrum illness and not a primary psychotic disorder (Sit et al. 2006; Chaudron and Pies 2003; Jones and Craddock 2002). In the absence of formal guidelines, treatment in clinical practice is typically based on the most prominent symptom dimensions. Benzodiazepines are used for insomnia and agitation, antipsychotics and mood stabilizers for psychotic and manic symptoms, and antidepressants for depressive symptoms.

During admission, women with a first-onset postpartum psychosis were treated according to a standardized treatment algorithm, as described previously (Bergink et al. 2015b). The first step in treatment involves benzodiazepines at bedtime for three days (step 1). The purpose of starting with an initial period of benzodiazepine monotherapy is to evaluate whether restoration of sleep results in clinical remission of manic and psychotic symptoms, as sleep loss has been considered an important etiological factor in postpartum psychosis (Sharma and Mazmanian 2003). For patients whose manic or psychotic symptoms persist after a few days of benzodiazepine monotherapy, the next recommended step involves antipsychotic medication (step 2). Although the efficacy of antipsychotics in the absence of mood stabilizers has been described in only three case reports, antipsychotics are frequently used worldwide as first-line treatment for patients with postpartum psychosis and mania (Doucet et al. 2011). Furthermore, antipsychotics are often considered the preferred pharmacological treatment option for acute mania outside the postpartum period (Cipriani et al. 2011). Our primary recommendation for antipsychotic treatment was haloperidol at 2–6 mg/day. Patients who experienced side effects were switched to an atypical antipsychotic. A subset of patients who had already been treated with an antipsychotic before admission (e.g., by acute services) were continued on the same antipsychotic they received before admission. If there was no significant clinical response after 2 weeks, adjunctive lithium was initiated (step 3). One small open-label study and a case report have suggested that the combination of lithium and antipsychotics is more effective than antipsychotic monotherapy in patients with postpartum psychosis (Targum et al. 1979; Silbermann et al. 1975). Furthermore, studies have demonstrated efficacy of lithium in the prevention of postpartum psychosis (Stewart et al. 1991; Cohen et al. 1995; Austin 1992; Bergink et al. 2012). Lithium dosing was achieved based on plasma level (target, 0.8–1.0 mmol/L).

After complete remission of symptoms, all women were advised to taper benzodiazepines to discontinuation. Women receiving antipsychotic monotherapy were advised to continue this treatment as maintenance therapy until nine months postpartum. Women who achieved clinical remission using both antipsychotics and lithium were advised to gradually taper off antipsychotic treatment, with maintenance lithium monotherapy until nine months postpartum. Lithium dosing for relapse prevention was achieved based on plasma level (target, 0.6–0.8 mmol/L).

### Longitudinal course of the illness

Four years postpartum, women were re-evaluated using the SCID (First et al. 1999). Women were not seen in between hospital discharge and follow-up for the purposes of this study. Recurrence was defined as the occurrence of any depression, (hypo)mania, psychosis or mixed state episode fulfilling DSM-IV criteria, admission to hospital or a restart of medication. All women with a recurrence were asked retrospectively about the timing of their episode, including whether this was in relation to a subsequent pregnancy. Additionally, we collected information on the timing of tapering or stopping medication if applicable. The patient’s medical records were consulted to validate the information.

Based on information collected at follow-up, women were categorized into one of two groups: (1) women with recurrence of non-postpartum mood or psychotic episodes within the follow-up period, or (2) women with mania/psychosis in the postpartum period and no mood or psychotic episodes outside the postpartum period during follow-up (vulnerability to affective psychosis only after childbirth).

### Statistical analysis

We summarize the longitudinal course of the illness per in Table 1. Differences between the two groups in terms of baseline demographic and clinical characteristics were assessed using Chi-squared and t-tests were appropriate (Table 1). A Kaplan–Meier Survival Curve of recurrence rates within the four-year follow-up period after first-onset postpartum psychosis was plotted. Additionally, we used a binomial logistic regression model to identify clinical predictors of postpartum psychosis group (recurrence of non-postpartum mood or psychotic episodes vs. mania/psychosis in the postpartum period only). Potential predictors of recurrence were based on the literature included admission length, maternal age, phenomenology of the index episode, and family history of psychiatric illness (Kapfhammer et al. 2014; Blackmore et al. 2013; Benvenuti et al. 1992). To improve power in the family history variable, depression and anxiety were combined into the category ‘depression or anxiety’ and postpartum depression and postpartum psychosis were combined into the category ‘postpartum psychiatric episode’. Mania/psychosis in the postpartum period only and psychiatric disorder with non-postpartum episodes were coded as 0 and 1 respectively. Results are presented in the form of odds ratios. All statistical analyses were performed using Stata/MP 15 (StataCorp 2017). Lastly, we explored whether the set of psychotic symptoms recognized as having special weight in the diagnosis of schizophrenia and schizoaffective disorder (thought echo, insertion, withdrawal, or broadcasting; passivity experiences; hallucinatory voices giving running commentary, discussing subject in third person, or originating in some part of the body; bizarre delusions; catatonia) was a precursor for a diagnosis within the psychotic disorder spectrum.

## Results

### Follow-up

Seventy-two women (67.9%) did not experience recurrence during the four-year follow-up. Two women (1.9%) experienced a recurrence exclusively following later pregnancies. Both of these women subsequently received a diagnosis of depression at follow-up (Table 1).

**Fig. 1:**
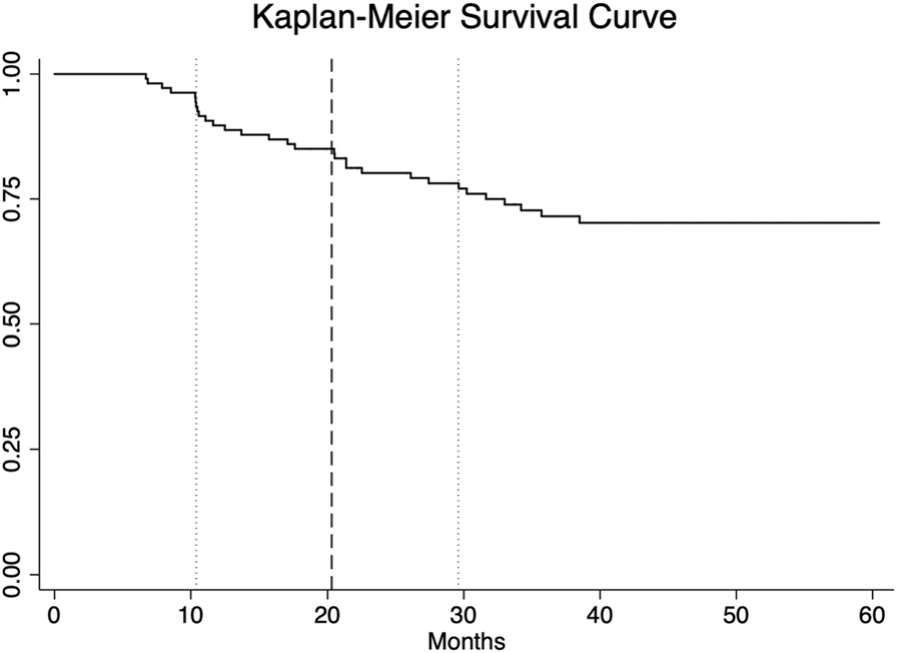
Kaplan–Meier Survival Curve of recurrence rates within the four-year follow-up period after first-onset postpartum psychosis. Median time to recurrence is represented by a dashed line, the interquartile range is represented by dotted lines

Thirty-four women (32.1%) experienced at least one additional episode outside of the postpartum period during follow-up. The median time to recurrence during follow-up in women with episodes outside of the postpartum period was 20.3 months (interquartile range IQR: 10.4–29.6) following initial hospitalization (Fig. 1). Of the thirty-four women who had at least one episode outside of the postpartum period, 14 experienced an episode of (hypo)mania (13.2% of the overall sample), 11 experienced a depressive/anxiety episode (10.4% of the overall sample), and nine experienced a psychotic episode with or without affective components (8.5% of the overall sample) within the follow-up period.

### Medication use

The majority of patients were treated with lithium (76.4%) and antipsychotics (83.0%) during their MBU admission. Over the course of the follow-up period, most women were able to successfully taper lithium: out of the 52 women who stopped lithium, five relapsed within six months of discontinuation (Table 1).

### Potential predictors of relapse

To identify potential clinical predictors of recurrence of non-postpartum mood or psychotic episodes, we carried out a logistic regression. We did not find significant predictors of recurrence outside the postpartum period (Table 2).

**Table 2:** Logistic regression analysis of the clinical predictors for recurrence of non-postpartum mood or psychotic episodes within the follow-up period

		ß	z	p	OR	OR 95% CI
Admission length		0.001	0.07	0.943	1.00	0.98; 1.02
Age		− 0.06	− 1.16	0.245	0.94	0.86; 1.04
Phenomenology at admission	Psychotic only	0.46	0.74	0.460	1.59	0.47; 5.42
	Depressed-psychotic	− 0.48	− 0.55	0.580	0.61	0.11; 3.47
	Manic with and without psychotic features	0.55	0.81	0.418	1.74	0.46; 6.61
Family history	Postpartum episode	− 1.65	− 1.50	0.133	0.20	0.02; 1.65
	Depression or Anxiety	0.17	0.36	0.717	1.18	0.48; 2.94
	Bipolar disorder	− 0.70	-0.94	0.345	0.50	0.12; 2.12
Lithium treatment of initial episode		0.21	0.35	0.727	1.24	0.37; 4.11
Antipsychotic treatment initial episode		0.26	0.42	0.677	1.30	0.38; 4.42

### Psychotic symptoms

Eight women (7.5%) in this cohort experienced first rank psychotic symptoms during their postpartum psychosis as measured by the BADDS. These include one or more of the schizophrenia-like symptoms, including thought insertion, withdrawal or broadcasting, passivity experiences, hallucinatory voices giving running commentary, discussing subject in third person and bizarre delusions. Of these eight women, five experienced no recurrence, while two women experienced (hypo)mania within the first year after their initial episode. One woman experienced depressive episodes both during a subsequent postpartum episode, as well as outside the postpartum period. None of the women with first-rank psychotic symptoms met criteria for a schizophrenia spectrum illness, including schizophreniform, schizophrenia, and schizoaffective disorder, during follow-up.

## Discussion

In this prospective longitudinal study, we investigated the long-term outcome of 106 women with postpartum psychosis over a four-year period. Over two thirds of the women included in this study did not have psychiatric episodes outside the postpartum period during follow-up. For the remaining subset of women (~ 32%), postpartum psychosis was part of a psychiatric disorder with a more disabling disease course and broader window of recurrence vulnerability, both in and outside of the postpartum period. This recurrence rate for mood or psychotic episodes outside the postpartum period is lower than the recurrence rate we recently reported in our meta-analysis (56.5%) (Gilden et al. 2020).

The differences between this study and our recent meta-analysis (Gilden et al. 2020) may be due to the differences in the follow-up period, which was four years in this study but ranged from 11 to 26 years in the meta-analysis. It is conceivable that recurrence rates increase with longer follow-up periods. The relatively lower relapse rates may also be attributed to preventive follow-up, including continued medication use, and specialized health care for these women in the current treatment setting (Bergink et al. 2011).

Of the 34 women with a recurrence, 14 experienced an episode of (hypo)mania (13.2% of the overall sample), 11 experienced a depressive/anxiety episode (10.4% of the overall sample), and nine experienced a psychotic episode with or without affective components (8.5% of the overall sample) within the follow-up period. Of the nine women who experienced a psychotic episode, four women met diagnostic criteria for schizoaffective disorder, four women met diagnostic criteria for brief psychotic disorder, and one met diagnostic criteria for psychotic disorder not otherwise specified. None of these women fulfilled diagnostic criteria for schizophrenia or schizophreniform disorder. Currently, the DSM-5 does not recognize postpartum psychosis (including psychotic, manic, psychotic depressed, or mixed episodes) as a distinct disease category (Kamperman et al. 2017). Women with psychotic symptoms without an affective component are currently diagnosed as either psychosis not otherwise specified, brief psychotic disorder or schizophreniform disorder, if schizophrenia-like symptoms are present. A primary diagnosis within the psychotic DSM may not be accurate.

The majority of women with postpartum psychosis have prominent manic or mixed affective features (Kamperman et al. 2017). Based on current best practice, these women are, therefore, diagnosed with bipolar disorder at the time of their first-onset postpartum psychosis. However, the fact that over 67% of our sample (and 43.5% in our recent meta-analysis (Gilden et al. 2020)) had no depressive, manic or psychotic recurrence outside the postpartum period raises questions about the validity of this approach. The diagnosis ‘bipolar disorder’ suggests a vulnerability to mood episodes at all times, not only during the postpartum period. Consequently, we believe a diagnosis of bipolar disorder should only be given following severe mood episodes outside of the postpartum period, either mania or depression. For women with vulnerability for episodes limited to the postpartum period, a distinct classification within the bipolar spectrum would be more accurate and reduce stigma.

To investigate predictive factors of episodes outside of the postpartum period, we assessed the association between various clinical and demographic characteristics with recurrence outcome. Unlike previous retrospective studies (Sit et al. 2006; Terp et al. 1999; Blackmore et al. 2013), we did not find that the length of the disease episode or a woman’s age were significantly associated with higher risk for developing a more severe psychiatric disorder with non-postpartum episodes. This may be due to our standardized treatment algorithm, as well as the lack of variance in maternal age in our sample. Moreover, phenomenology of the index episode was also not predictive of the disease course, but this may be attributed to a lack of statistical power. The risk of recurrence for women with mania (both with or without psychotic symptoms) or psychosis without affective symptoms was very similar. Surprisingly, a schizophrenia-like presentation was neither predictive of recurrence, nor of receiving a schizoaffective diagnosis during follow-up. In line with prior longitudinal studies (Kapfhammer et al. 2014; Terp et al. 1999; Benvenuti et al. 1992; Kirpinar et al. 1999; Rohde and Marneros 1993; Schöpf and Rust 1994; Videbech and Gouliaev 1995), we found that the vast majority of non-postpartum episodes during follow-up occurred within the bipolar spectrum. In our cohort, none of the women received a diagnosis of schizophrenia during follow-up, similar to most prior studies, except Kirpinar et al. (Kirpinar et al. 1999), who found a relationship between postpartum psychosis and a diagnosis of schizophrenia during follow-up.

Understanding who is at risk of a mood or psychotic disorder during follow-up, and whose vulnerability is limited to the postpartum period, is particularly important in guiding treatment decisions including long-term pharmacotherapy. Unfortunately, no biomarkers are currently available to help guide these decisions. In clinical practice, this means that long-term monitoring is warranted for everyone with postpartum psychosis. Another reason for long-term monitoring are the high suicide rates during follow-up, reported by other studies (Kapfhammer et al. 2014; Schöpf and Rust 1994; Videbech and Gouliaev 1995).

Our findings must be interpreted in light of a number of limitations. Firstly, our sample size was relatively small. Nevertheless, this is the largest prospective longitudinal study of women with postpartum psychosis to date. Secondly, this is a naturalistic study, rather than a randomized control trial, in which patients’ preferences may have influenced treatment decisions. Due to the low incidence of postpartum psychosis, a randomized control trial for its treatment would be challenging, and no such trial has been published (Bergink et al. 2016). Lastly, our cohort was recruited from a single, inpatient site in the Netherlands. Patients were more highly educated and more likely to be partnered/married than the general population, potentially limiting the generalizability of our findings.

## Conclusion

In this prospective longitudinal study of first-onset postpartum psychosis, we investigated the long-term outcomes of 106 women with postpartum psychosis over a four-year follow-up. We found that for the majority of women with first-onset postpartum psychosis, the risk of illness was limited to the period after childbirth. For the remaining women, postpartum psychosis was part of a mood or psychotic disorder with severe non-postpartum recurrence, mainly in the bipolar spectrum. No clinical predictors of a woman’s risk of severe episodes outside the postpartum period were found. Our findings add to previous evidence suggesting a fundamental link between postpartum psychosis and bipolar disorder, which may represent two distinct diagnoses within the same spectrum.

## Supplementary Information

**Additional file 1: Table A1**. Baseline and clinical characteristics of the women lost to follow-up.

## Data Availability

The dataset analysed during the current study is not publicly available due to ongoing data collection but may be available from the corresponding author on reasonable request.

## Abbreviations

95%CI: 95% Confidence interval
BADDS: Bipolar Affective Disorder Dimension Scale
D: BADDS Depression dimension
M: BADDS Mania dimension
P: BADDS Psychosis dimension
DSM: Diagnostic and Statistical Manual of Mental Disorders
Erasmus MC: Erasmus Medical Centre
ICD-10: International Classification of Diseases, Tenth Revision
IQR: Interquartile range
MBU: Mother-Baby Unit
mmol/L: Millimoles per liter
NOS: Not otherwise specified
OR: Odds ratio
SCID: Structured Clinical Interview for DSM
