# Supplementary material for: Long-term outcome of postpartum psychosis: a prospective clinical cohort study in 106 women
Source: Int J Bipolar Disord. 2021 Oct 28;9:31. doi: 10.1186/s40345-021-00236-2 (PMC8554899; doi:10.1186/s40345-021-00236-2)
Supplement: Supplementary file 1 — Additional file 1: Table A1. Baseline and clinical characteristics of the women lost to follow-up. [file 40345_2021_236_MOESM1_ESM.docx]

**Appendix A.**

**Table A1**. Baseline and clinical characteristics of the women lost to follow-up

|  | | **Lost to follow-up**  (n=6) |
| --- | --- | --- |
| **Mean age in years (SD)** | | 32.9 (7.8) |
| **Native country % (n)** | Netherlands | 100 (6) |
| **Marital status % (n)** | Married or in relationship | 100 (6) |
| **Education % (n)** | No education | - |
|  | Primary school | - |
|  | Secondary school | 33.3 (2) |
|  | Vocational training | 33.3 (2) |
|  | Higher education | 33.3 (2) |
| **Parity % (n)** | 1 | 100 (6) |
| **Primary psychiatric history % (n)** | None | 100 (6) |
| **Family history of psychiatric disorders %^†^ (n)** | None | 33.3 (3) |
|  | 1^st^ degree relative with depression or anxiety | 33.3 (3) |
|  | 1^st^ degree relative with postpartum psychiatric episode | - |
|  | 1^st^ degree relative with bipolar disorder | 11.1 (1) |
| **Mean length of initial hospital admission in days (SD)** | | 78.2 (35.9) |
| **Phenomenology** | Manic with and without psychotic features | 33.3 (2) |
|  | Psychotic only | 33.3 (2) |
|  | Depressed-psychotic | 33.3 (2) |
|  | Manic-depressed (mixed) | 0 (0) |
| **Lithium treatment during admission % (n)** | No | 0 (0) |
|  | Yes | 100 (6) |
| **Suicide % (n)** | | 16.7 (1) |
